# Systematically Controlling for the Influence of Age, Sex, Hertz and Time Post-Whole-Body Vibration Exposure on Four Measures of Physical Performance in Community-Dwelling Older Adults: A Randomized Cross-Over Study

**DOI:** 10.1155/2011/747094

**Published:** 2011-09-29

**Authors:** Harold L. Merriman, C. Jayne Brahler, Kurt Jackson

**Affiliations:** Physical Therapy Program, Department of Health & Sport Science, University of Dayton, Dayton, OH 45469-2925, USA

## Abstract

Though popular, there is little agreement on what whole-body vibration (WBV) parameters will optimize performance. This study aimed to clarify the effects of age, sex, hertz and time on four physical function indicators in community-dwelling older adults (*N* = 32). Participants were exposed to 2 min WBV per session at either 2 Hz or 26 Hz and outcome measures were recorded at 2, 20 and 40 min post-WBV. Timed get up-and-go and chair sit-and-reach performances improved post-WBV for both sexes, were significantly different between 2 Hz and 26 Hz treatments (*P* ≤ 0.05) and showed statistically significant interactions between age and gender (*P* ≤ 0.01). Counter movement jump and timed one-legged stance performances showed a similar but non-significant response to 2 Hz and 26 Hz treatments, though male subjects showed a distinct trended response. Age and gender should be statistically controlled and both 2 Hz and 26 Hz exert a treatment effect.

## 1. Introduction

Whole-body vibration (WBV) is becoming a popular modality used to improve functional mobility and balance in older adults as well as to enhance muscle performance in elite athletes [1–5]. WBV involves exposing the entire body to vibration as the subject stands on a vibrating platform. More commonly, WBV has been used to improve physical competitive performance of athletes [2, 6–10]. Such improvements in muscle strength and power after WBV may be related to an increase in neuromuscular activation during and following WBV. One possibility for WBV action is through the vibratory stretch reflex in which the mechanical vibration elicits a myotactic stretch reflex mediated by the muscle spindle and Ia-afferents [11]. Other possible mechanisms of beneficial WBV action include increasing neuromuscular efficiency, enhancing postural control, increased gravitational force, and improving muscle quality which is complicated by the complex interplay of agonists and antagonists [4, 5, 7, 8, 12–14].

Despite the growing use of WBV for older adults in both community and clinical settings, little is known about the optimal and safe WBV parameters (Hz, amplitude and duration) that should be used for training. Published research studies to date have been quite variable and have used a wide range of WBV parameters with diverse subject populations [2, 3, 5, 14–16]. While studies involving older adults have generally shown improvements in bone density, muscle performance, functional mobility, and balance, no studies have systematically evaluated the effects of specific WBV treatment parameters on these outcome measures and how factors such as age and sex might also influence responses to WBV [3].

Another weakness of most WBV research is the difficulty of providing a true control or placebo condition. Developing a proper control is one of the most challenging aspects of WBV study design due to the nature of the intervention which provides a strong physical and sensory stimulus. Some WBV studies have used a 0 Hz control with subjects standing on a nonvibrating WBV unit [17, 18]. With this method, subjects easily figure out when they are or are not receiving WBV even if they listen to a tape-recorded sound of a vibrating WBV unit [19]. An ideal control is to engineer a placebo unit that makes the same sounds but does not vibrate as a regular WBV unit as was developed by Rubin et al. [20] to study low-magnitude WBV. Most other WBV studies, like this present study, have used commercial WBV units, where ideal control options are limited.

Given the weaknesses of current WBV research, the primary purpose of this study was to systematically determine the individual and collective influences of age, sex, hertz, and time post-whole-body-vibration exposure on four measures of physical performance in community-dwelling older adults. A secondary objective was to evaluate the potential of using a low-frequency (2 Hz) vibration setting as a possible control or placebo condition. Additionally, we evaluated potential safety concerns by comparing the WBV dosages used in this study with the current International Organization for Standardization (ISO) recommendations [12].

## 2. Methods

### 2.1. Subjects

**Figure 1:**
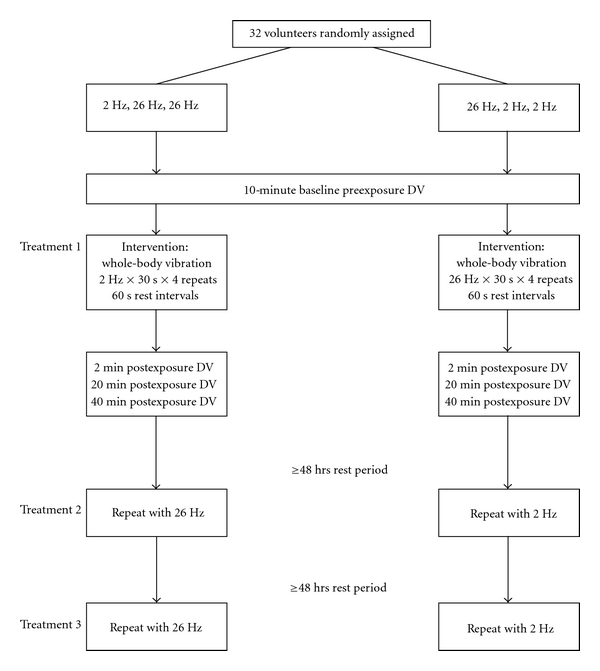
A schematic presentation of the experimental procedures. Inclusion criteria were ≥65 years old, live in the community, and able to comfortably walk at least 150 feet without an assistive device. Exclusion criteria were acute thrombosis, acute inflammation, acute tendinopathy, fresh fractures, gallstones, implants (pacemaker, breast implant, buttock implant, screws, pins, pumps, wires), recent surgery, acute hernia, acute discopathy, acute migraine, fresh wound/scar, epilepsy, total knee replacement, total hip replacement, infectious disease, uncontrolled diabetes, neuromuscular disease, and osteoporosis. Abbreviations: DV, dependent variables; TGUG, timed get up and go; OLST, one-legged stance test; CMJ, counter movement jump; CSR, chair sit and reach.

Older adult subjects were recruited by public advertising in the Dayton Ohio community. Thirty-five individuals expressed interest in the study and were assessed for eligibility by a licensed physical therapist for this particular study. Thirty-two subjects (10 males, 22 females; mean age ± SD, males: 72.3 ± 6.5 y, females: 71.7 ± 6.0 y, total: 71.9 ± 6.1 y) met the inclusion/exclusion criteria (Figure 1). Subjects' weight for males was 90.5 ± 10.7 kg and for females was 66.4 ± 9.9 kg, while subjects' height for males was 175.1 ± 4.7 cm and for females was 161.3 ± 7.0 cm.

Inclusion criteria were ≥65 years old, live in the community, and able to comfortably walk at least 150 feet without an assistive device. Exclusion criteria were acute thrombosis, acute inflammation, acute tendinopathy, fresh fractures, gallstones, implants (pacemaker, breast implant, buttock implant, screws, pins, pumps, wires), recent surgery, acute hernia, acute discopathy, acute migraine, fresh wound/scar, epilepsy, total knee replacement, total hip replacement, infectious disease, uncontrolled diabetes, neuromuscular disease, and osteoporosis. All study participants gave their informed written consent before starting the study, and the protocol was approved by the Institutional Review Board of the University of Dayton. The procedures in this study followed approved protocol.

### 2.2. Design

This study used a randomized three-period cross-over study with repeated measures design (Figure 1).

### 2.3. Equipment

Subjects were exposed to WBV using a rotational tilt vibration unit manufactured by Maxuvibe (Model: Pro, Perfect Fitness BV, Eindhoven, The Netherlands) that oscillates over a user-adjusted frequency of 0 to 30 Hz with a variable amplitude from 0–12.5 mm. A biaxial accelerometer (ADXL 320 EB, Analog Devices Inc., Norwood, Mass, USA) was used in separate testing not involving subjects to determine the WBV machine's parameters according to the recommendations of the International Society of Musculoskeletal and Neuronal Interactions [21]. Jump height was calculated using a contact platform (Just Jump, MF Athletic Company, Cranston, RI, USA).

### 2.4. Protocol

This single-blinded three-period cross-over study (ABB/BAA) consisted of two treatment arms (Figure 1). Using a random number generator, subjects were assigned to receive either 2 Hertz (A) or 26 Hertz (B) as their first treatment. In the ABB/BAA design, following the first treatment, the subjects cross over to the other treatment condition-for their remaining two trials. A major strength of a cross-over design is that each participant serves as their own control. An additional design strength is gained with the third-period variant of cross-over designs because the effects of carryover (the effect of going from A to A or B to B) can be quantified and statistically controlled if necessary. 

There was a minimum of 48 hours between each of the three WBV sessions (ABB or BAA). The subjects were told that they would receive WBV at different frequencies prior to testing but were not told which frequency they received during the test. For a given subject, one investigator set the WBV parameters, and a different investigator recorded the outcome measures. Subjects were not exposed to a warm-up period prior to testing.

Subjects were tested by a blinded examiner (to WBV frequency) for the following four outcome measures: (1) functional mobility by the timed get up-and-go (TGUG) test [22], (2) static balance by the timed one-legged stance test (OLST) with eyes closed [23, 24], (3) muscle power performance by the vertical countermovement jump ( CMJ) test that measures height jumped [25], and (4) lower body flexibility by the chair sit-and-reach (CSR) test [26]. Subjects repeated each outcome measure twice at 10 minutes before and at 2, 20, and 40 minutes after WBV intervention. The average value of these two repeated measures was subsequently used in data analysis. Subjects wore a gait belt for safety during WBV exposure and outcome measure testing, and an investigator spotted the subject as appropriate.

**Figure 2:**
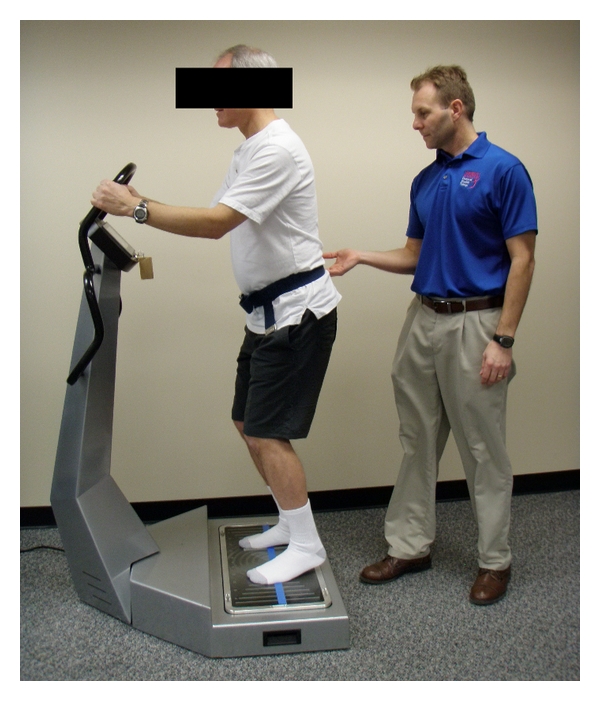
WBV setup. Subjects were exposed to 4 bouts of 30 seconds of WBV either at 2 Hz or 26 Hz interspersed with a 60-second rest break between bouts.

While standing on the WBV unit, subjects wore no footware and flexed their knees and hips to about 30 degrees with heels off the platform and their weight on their forefoot (Figure 2). To increase safety during WBV, subjects held onto a support bar with both hands. WBV was applied at either 2 Hz or 26 Hz for 4 bouts of 30 seconds with a 60-second standing rest break in between bouts which is a common exposure time frame used for rotational-tilt WBV protocols involving older adults. For post-WBV testing, subjects remained seated except during the outcome measure testing. 

### 2.5. Statistical Analysis

Descriptive statistics were calculated for subject demographic data and for all outcome measures. Due to the three-period cross-over study design, each subject completed a total of 3 trials consisting of 1 trial at an initial vibration frequency and 2 trials at the other vibration frequency. Repeated measures generalized linear model (RM GLM) tests were completed on data collected ten minutes prior to each treatment session to determine if any baseline values were significantly different at baseline. This analysis was completed in order to establish that all treatment sessions were starting at comparable baseline levels and to determine if there had been any lasting effect from a preceding treatment. A major strength of the three-period cross-over design is its ability to determine if there is a carry-over or treatment effect when a subject receives more than one treatment at the same or different vibration frequencies, respectively. There were no significant differences at baseline for any variables. Paired sample *t*-tests were completed on the two observations collected for the second treatment condition (AA or BB) to determine if the two observations were statistically significantly different from each other to determine if there was a carry-over effect. If not, the data for two trials completed at the same WBV frequency could be averaged.

Using these data, RM GLM tests were completed for each outcome measure with the data collected for each subject at 2 and 26 Hz serving as the repeats. Subject age was entered as a covariate, and gender and time of measurement (−10, 2, 20, and 40 minutes relative to WBV treatment time) were included as factors. Estimated marginal means were calculated for each dependent variable to estimate the population marginal means after adjusting for the covariate of age. The RM GLM testing accounts for the variance due to a significant covariate by mathematically adjusting the measured mean values of any given variable to what they would be if all subjects were the same on that particular variable. Alpha was set at 0.05 for all testing.

## 3. Results

### 3.1. WBV Machine Parameters

Using the biaxial accelerometer on this particular WBV machine, the measured Hz at the WBV machine settings of 2 Hz and 26 Hz was found to be 1.80 Hz and 25.68 Hz, respectively. Peak-to-peak displacement was 3.87 mm, while peak acceleration in *g* was 0.014 at 2 Hz and 5.14 at 26 Hz. Finally, estimated vibration dose value (eVDV) in *g* over the 120 seconds of WBV total exposure was calculated to be 0.05 at 2 Hz and 16.84 at 26 Hz.

### 3.2. Descriptive and Research Design Statistics

All 32 participants completed the study without any adverse events. All 32 subject completed every aspect of testing, with the exception of one individual who was not able to participate in the third data collection session due to having elevated blood pressure prior to initiating the third WBV protocol. For this subject, data for the second WBV treatment condition was comprised of a single bout at that vibration frequency, rather than an average of two bouts at that frequency. As such, the table and all Figures included in this report represent data for all 32 subjects. There were no statistically significant differences between baseline measurements between the two study groups; therefore, it was concluded that the two groups were similar enough to be compared. There were no significant differences in any measures between the two repeats at the second vibration condition (AA or BB); therefore, the data for the two observations were averaged. Means, standard errors, and 95% confidence intervals for all four outcome measures are found in Table 1.

### 3.3. Effects of WBV on Specific Outcome Measures

#### 3.3.1. TGUG

**Figure 3:**
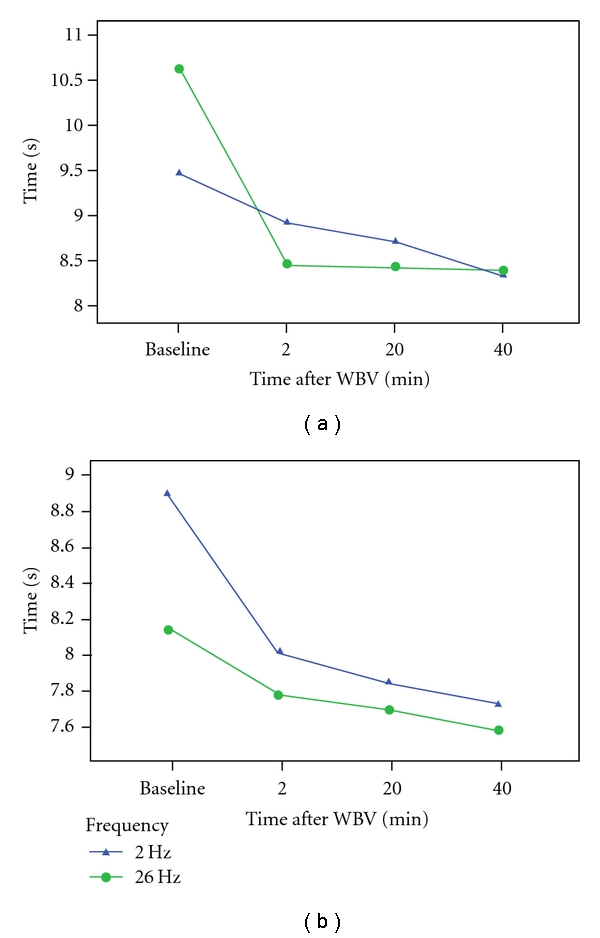
Time course of timed get up-and-go test in males (a) and females (b) at 2 Hz and 26 Hz WBV.

TGUG performances improved from baseline to 2, 20, and 40 minutes after treatment for both genders and in response to both 2 and 26 Hz treatments (Figure 3). As a repeated measure, TGUG performances were significantly different for the study subjects between 2 and 26 Hz WBV (*P* ≤ 0.01). There were statistically significant interactions between TGUG and age (*P* ≤ 0.01) and between TGUG and gender (*P* ≤ 0.01). The significant interaction between TGUG and gender is depicted in Figure 3 where, compared to baseline, males' performance improved most following the 26 Hz treatment, while females' performance improved most following the 2 Hz treatment. Additionally, there was an overall trend for males to make greater improvements compared to females, regardless of the treatment condition. An independent samples *t*-test revealed that baseline TGUG performances were not statistically significantly different at baseline (*P* = 0.822) between the groups receiving 2 Hz first and those receiving 26 Hz first.

#### 3.3.2. CSR

**Figure 4:**
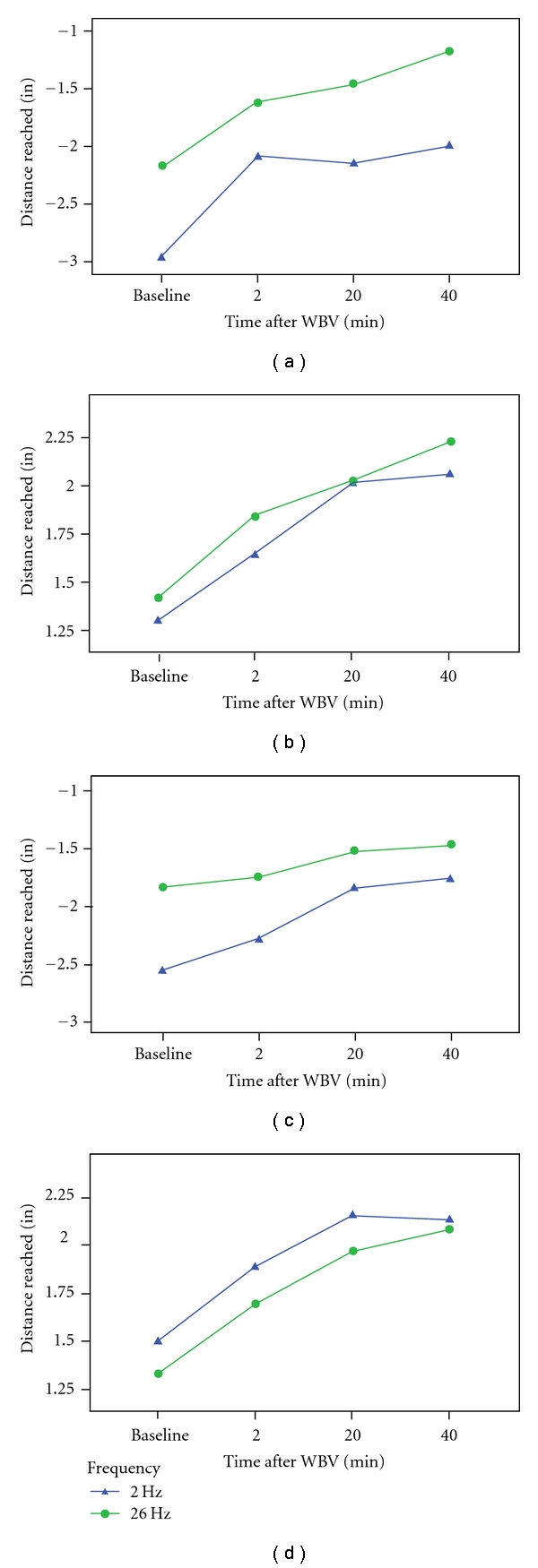
Time course of chair sit and reach (CSR) on the left side in males (a) and females (b) and CSR on the right side in males (c) and females (d) at 2 Hz and 26 Hz WBV.

Right and left CSR measures improved from baseline to 2, 20, and 40 minutes after treatment for both genders and to both 2 and 26 Hz treatments (Figure 4). As a RM, CSR performances were significantly different between 2 and 26 Hz treatment for the study subjects (left CSR *P* ≤ 0.01; right CSR *P* ≤ 0.05). There were statistically significant interactions between CSR (both left and right) and age (*P* ≤ 0.01) and between CSR (both left and right) and gender (*P* ≤ 0.01). The significant effect of age was statistically controlled as a confounder in the study. Although both genders improved with both treatments, the performances were quite different among the genders. Males, on average, were much less flexible than were females, and males tended to respond more favorably to the 26 Hz treatment relative to the 2 Hz treatment, whereas females showed very similar responses to both 2 and 26 Hz treatments.

#### 3.3.3.  CMJ, OLST

 CMJ and OLST (right and left) were not significantly different in response to 2 versus 26 Hz treatments. In male subjects, we also observed a trend of 2 Hz increasing and 26 Hz decreasing CMJ height following WBV exposure.

## 4. Discussion

### 4.1. WBV Study Covariates

RM GLM testing revealed that subject age was the most significant single source of variance in the current study. Thus, mean values for the outcome measures were mathematically adjusted for the mean age of the study sample, which was 71.88 years. Specifically, age was a significant source of confound on every measured outcome (TGUG, CSR, right and left CSR, and OLST; *P* ≤ 0.01 for each). Age had a linear association with subject performance, and subject performance declined with advancing age. To date, WBV studies in older adults have not controlled for age which is an inherit weakness in these studies [3–5, 14].

Gender was also found to be a significant source of variance on all outcome measures (*P* ≤ 0.01 for each) except for right OLST (*P* = 0.053). Time of measurement was not a significant source of variance in the measured outcomes. However, Figures 3 and 4 display the mean values for all outcome measures broken out by the time of their measurement to convey the time course of the responses to WBV in the study.

TGUG and right and left CSR performances (see Figures 3 and 4) were significantly different between the two different vibration frequencies (*P* ≤ 0.01 for TGUG and left CSR; *P* ≤ 0.05 for right CSR). Right and left OLST and CMJ performances, on the other hand, were not significantly different between 2 and 26 Hz treatment conditions. Male and female data are represented individually because of the significant impact gender had on subject performances.

### 4.2. Possible Sex Differences in Response to WBV

Most WBV studies using older adult subjects have either predominately or exclusively used female subjects [3–5, 14]. This gender bias may be due to the desire to study postmenopausal women as well as the higher percentage of females reaching older age. Only a few WBV studies have included a near majority or majority of older adult male subjects [18, 27, 28].

Whether men and women of any age might respond differently to WBV has largely remained unexplored. Torvinen et al. [29] found no gender differences when studying the effects of WBV on muscle performance and body balance in a small group of young adult subjects (*n* = 16) equally divided among men and women. However, this present study provides evidence that WBV may have different effects on males and females. Clearly, more studies involving male and female older adults are needed to determine if this observation has merit. One may need to be guarded when reading study results where age and gender were not statistically controlled.

### 4.3. 2 Hz WBV Frequency Serves as a Treatment and Not a Sham

Developing a proper control is one of the most challenging aspects of WBV study design especially where higher-magnitude commercial WBV units are used. A number of strategies such as having subjects stand on a non-vibrating WBV unit [17, 18] or listen to tape-recorded vibration sounds [19] have been employed with variable effectiveness as previously mentioned.

In this study, we investigated the feasibility of using 2 Hz as a potential control. Preliminary data collected in our lab using surface electromyography of the vastus medialis showed little or no increased WBV activity at 2 Hz versus 0 Hz. Based on our repeated measure study design which included a pretest 0 Hz collection point for each of the three sessions, we wanted to investigate if 2 Hz might serve as an appropriate control. To date, the effects of lower vibration frequencies in the 2–6 Hz range have not been studied extensively though some benefits of WBV in this range have been observed [30–32].

This study showed that 2 Hz should not serve as a control or sham treatment because it elicited a treatment effect in several cases. One advantage of using 2 Hz is that this lower frequency is more comfortable and easily tolerated, especially during the initial exposure periods. Given these potential benefits of 2 Hz, it was intriguing to note that CMJ height of male subjects tended to increase at 2 Hz but decrease at 26 Hz over time. A similar but less obvious trend was observed in female subjects.

### 4.4. Optimal Safe WBV Parameters and Machine Type

There is considerable debate concerning what constitutes safe WBV exposure times in a clinical setting (i.e., 1–5 minutes/day) versus chronic occupational exposure. Abercromby et al. [12] observed that exposure to clinical doses of WBV (up to 10 min/day, 30 Hz, 4 mm amplitude) often exceeds the ISO recommendations for chronic vibration exposure. The ISO recommended eVDV exposure maximum of 17 compares favorably with the present study's eVDV exposures of 0.014 at 2 Hz and 16.84 at 26 Hz. Some evidence suggests that exposure to WBV for >30 sec results in a decrease and not an increase in voluntary muscle activation [4]. Therefore, a prudent approach would be to determine the minimum WBV exposure that would still produce the desired clinical outcome.

It is also important to avoid harmful resonant frequencies which can result in nonlinearity of WBV transmission [33]. For example, the human body's internal organs vibrate in a frequency range of ~5 to ~20 Hz, and the eyeball has a resonance frequency from 20 to 21 Hz [34]. In an erect standing posture, the hip exhibits maximal resonance at 17 Hz, but transmissibility of WBV decreases markedly with the knees flexed 20 degrees [35]. Crewther et al. [36] found that *g* forces associated with subjects standing erect were greatest at 20 Hz as compared to 10 Hz and 30 Hz and that significant damping of *g* forces occurs as the distance from the vibration plate increases. Kiiski et al. [37] investigated vertical WBV with subjects standing upright. They found that peak acceleration was substantially amplified at the ankle, knee, hip, and spine as compared to peak acceleration produced at the WBV platform. The study subjects reported universal discomfort at 20 Hz and 25 Hz with an amplitude of ≥0.5 mm. Subjects in this present study assumed a more flexed lower extremity posture, which is known to transmit less vibration than an erect posture [5, 35].

The nature of vibration produced by the WBV machine is also an important safety consideration. Abercromby et al. [12] compared the effects of side-alternating WBV and vertical WBV on head acceleration (Ha_rms_). Side-alternating WBV exhibited decreased Ha_rms_ values, and the lowest of these Ha_rms_ values were found when the subjects' knees were flexed to 26–30 degrees (similar to flexion values used in this study). No adverse events were reported in this study. While a few subjects reported an initial adjustment period to get used to the sensation of the higher 26 Hz frequency, most patients found both 2 Hz and 26 Hz treatment to be enjoyable.

### 4.5. Study Limitations

Subjects were blinded to WBV frequencies but could easily guess which treatment they were receiving. A potential limitation was assuming that 2 Hz would be a sham condition when it appeared to exert an affect. This limitation suggests that inclusion of an actual 0 Hz treatment condition may be indicated.

## 5. Conclusions

In this study of community-ambulatory aging adults, age was a significant source of confound on all dependent variables. Gender differences were observed in the outcome measures, but the effects of WBV were not universally significant; therefore, gender in at least this study needed to be controlled. This study provides evidence that both age and gender are covariates and should be statistically controlled in older adults. In addition, 2 Hz as well as 26 Hz had a treatment effect on most of the dependent variables. Therefore, lower frequencies such as 2 Hz may lead to a more tolerable and safer WBV experience.

## Figures and Tables

**Table 1:** Means, standard errors and 95% confidence intervals for 4 outcome measures in 32 community-dwelling older adults (mean age 72).

Test	Hz	Mean	Std. error	95% confidence interval	
				Lower bound	Upper bound
TGUG (sec)	2* 26	8.502 8.404	0.172 0.250	8.161 7.908	8.843 8.900
CSR left (cm)	2* 26	−0.274 0.139	0.310 0.319	−0.889 −0.494	0.340 0.771
CSR right (cm)	2** 26	−0.097 0.066	0.303 0.305	−0.697 −0.538	0.502 0.669
CMJ (in)	2 26	8.919 9.295	0.193 0.188	8.538 8.922	9.300 9.667
OLST left (sec)	2 26	5.280 6.566	0.609 0.832	4.075 4.917	6.486 8.214
OLST right (sec)	2 26	7.066 6.595	0.944 0.882	5.197 4.847	8.934 8.342

Abbreviations: TGUG, timed get up and go; CSR, chair sit and reach, CMJ, counter movement jump; OLST, one-legged stance test. *Means are significantly different (*P* ≤ 0.01). **Means are significantly different (*P* ≤ 0.05).
